# Bio-Inductive Materials in Direct and Indirect Pulp Capping—A Review Article

**DOI:** 10.3390/ma13051204

**Published:** 2020-03-07

**Authors:** Marta Kunert, Monika Lukomska-Szymanska

**Affiliations:** Department of General Dentistry, Medical University of Lodz, 251 Pomorska St., 92-213 Lodz, Poland; marta.kunert@stud.umed.lodz.pl

**Keywords:** direct pulp capping, indirect pulp capping, ProRoot MTA, MTA Angelus, retroMTA, biodentine, theraCal LC, ACTIVA BioACTIVE, vital pulp therapy

## Abstract

The article is aimed at analyzing the available research and comparing the properties of bio-inductive materials in direct and indirect pulp capping procedures. The properties and clinical performances of four calcium-silicate cements (ProRoot MTA, MTA Angelus, RetroMTA, Biodentine), a light-cured calcium silicate-based material (TheraCal LC) and an enhanced resin-modified glass-ionomer (ACTIVA BioACTIVE) are widely discussed. A correlation of in vitro and in vivo data revealed that, currently, the most validated material for pulp capping procedures is still MTA. Despite Biodentine’s superiority in relatively easier manipulation, competitive pricing and predictable clinical outcome, more long-term clinical studies on Biodentine as a pulp capping agent are needed. According to available research, there is also insufficient evidence to support the use of TheraCal LC or ACTIVA BioACTIVE BASE/LINER in vital pulp therapy.

## 1. Introduction

The major challenge for the modern approach in restorative dentistry is to induce the remineralization of hypomineralized carious dentine, and therefore, protecting and preserving the vital pulp. Traditionally, deep caries management often resulted in pulp exposure and subsequent root canal treatment. The promotion of biologically-based treatment strategies has been advocated for partial caries removal aimed at avoiding carious pulp exposure. Indeed, recent consensus reports have stated that the complete or nonselective carious removal is now considered overtreatment [1]. Management strategies for the treatment of the cariously exposed pulp are also shifting with avoidance of pulpectomy, claiming the superiority of vital pulp treatment (VPT) techniques such as pulp capping, partial and complete pulpotomy [2]. This constant turn toward a conservative and minimally invasive approach is strongly associated with the fact that the emphasis of the profession is also slowly shifting to care for the senior adult segment of the population [3,4]. Under these circumstances, retaining natural teeth is of paramount importance. All of these changes occur in a strong correlation with development of so-called bioactive dental materials [5,6]. Broadly speaking, the bioactivity of a restorative material usually denotes that it has a biological effect or is biologically active. This characteristic refers to the potential to induce specific and intentional mineral attachment to the dentine substrate [7]. In terms of restorative dentistry, bioactive material is described as “one that forms a surface layer of an apatite-like material in the presence of an inorganic phosphate solution” [8]. Therefore, the remineralization of demineralized dentine is the process of restoring minerals through the formation of inorganic mineral-like matter [9].

Six different bioactive materials have been described and compared: four calcium-silicate cements—ProRoot MTA (Dentsply Sirona, York, PA, USA), MTA Angelus (Angelus, Londrina, Brazil), RetroMTA (BioMTA, Seoul, Korea) and Biodentine (Septodont, Saint-Maur-des-Fossés, France); a light-cured calcium silicate-based material TheraCal LC (Bisco, Schaumburg, IL, USA); and a resin-modified glass-ionomer (RMGIC) with improved resilience and physical properties—ACTIVA BioACTIVE (Pulpdent Corporation, Watertown, MA, USA) [10].

The article is aimed to review the properties of bio-inductive materials applied for direct and indirect pulp capping and to compare their clinical performances.

## 2. Vital Pulp Treatment (VPT) in Deep Caries and Management of Pulp Exposures 

The indirect pulp capping (IPC) procedure is generally used in deep cavity preparations, with or without carious dentine remaining which is in close proximity to the pulp but showing no visible pulp exposure [11,12]. According to the latest (2019) European Society of Endodontology (ESE)-approved definitions and terminology, IPC due to removal of both soft and firm carious dentine until hard dentine is reached, is nowadays considered aggressive, and in many cases may be acknowledged as overtreatment [13].

One-stage selective carious-tissue removal includes the application of a biomaterial onto a dentine barrier after either firm or soft dentine is left only on the pulpal aspect of the cavity, whilst peripheral carious dentine is removed down to hard dentine on the same visit permanent restoration is being placed. On the other hand, stepwise excavation involves re-entry after 6–12 months after application of a biomaterial in an indirect, two-stage, selective carious-tissue removal technique. The first stage involves selective carious removal to soft dentine, to an extent that facilitates proper placement of a temporary restoration, and the second stage includes removal to firm dentine. Eventually, final placement of a permanent restoration is performed. Tooth disinfection with NaOCl or chlorhexidine digluconate 2% solution, as a less aggressive alternative, sterile technique, and rubber dam use, are recommended at all times throughout the management of deep caries whether the pulp is exposed or not [13,14,15,16,17,18].

According to recent position statement of the ESE, deep caries management should focus on avoidance of pulp exposure by the means of choosing selective one-stage carious-tissue removal or stepwise excavation treatment, rather than redefined indirect pulp capping procedure [13].

Direct pulp capping (DPC) treatment is used when the vital asymptomatic pulp is visibly exposed due to caries or trauma, or due to a misadventure during tooth preparation or caries removal [7]. It includes the application of a biomaterial directly onto the exposed pulp, followed by immediate placement of a permanent restoration. An enhanced protocol should be used, including magnification, a disinfection irrigant and preferably the application of a hydraulic calcium silicate cement, as there is sufficient evidence to substantiate its use in VPT.

In contrast to pulp capping procedures, which does not involve any pulp tissue removal, partial pulpotomy removes 2–3 mm of the pulp tissue at site of exposure. In practice this technique is used for removing the superficial layer of infected tissue in case of asymptomatic carious pulp exposure or when pulp has been exposed to the oral environment. The pulp capping procedures protects the tissue, but may not reverse a superficial inflammatory processes; therefore, it is recommended that 2–3 mm of tissue is removed in a partial pulpotomy procedure. The question about choosing the proper treatment arises when we take into consideration that visual analysis to distinguish between inflamed and noninflamed pulp tissue may not be sufficiently accurate [19]. If hemostasis, as another factor indirectly indicating state of the pulp, cannot be controlled after 5 min, further caries excavation is necessary after exposure; in that case partial or even full pulpotomy may be preferable.

## 3. Materials Used in Direct and Indirect Pulp Capping

Historically, calcium hydroxide (Ca(OH)_2_) has been considered the gold standard. Long term clinical observations of calcium hydroxide are incomparable to any other bioactive material, as the first reports of successful pulpal healing using Ca(OH)_2_ were published between 1934 and 1941 [20]. However, it still has several drawbacks: insufficient adherence to dentinal walls, multiple tunnel defects in the induced dentin bridges [21], poor sealing ability, dissolution over time [22] and lack of antibacterial properties. Long-term clinical studies showed success rates with calcium hydroxide pulp capping on carious exposures to be highly variable, generally unpredictable and often unsuccessful [23]. Indeed, calcium hydroxide no longer seems to be the best possible material of choice [24]. Due to its high basicity, calcium hydroxide in direct contact with the pulp locally destroys a layer of pulp tissue, and thus creates an uncontrolled necrotic zone. This necrotic layer induces an inflammatory reaction which persists in time, or leads to formation of intra-pulpal calcifications [25]. However, it is Ca(OH)_2_ high solubility that is the major disadvantage of its use as a pulp capping agent. The dissolution of the material within two years after application and the formation of defects in reparative dentin underneath the capping material are responsible for failing to provide a permanent seal against bacterial infection.

Nowadays, calcium hydroxide is being displaced by new generations of materials which result in more predictable clinical outcomes; namely, calcium silicate materials (CSMs) and RMGIC [26,27]. The increasingly predominant role of CSMs could be explained by their high biocompatibility, intrinsic osteoconductive activity and ability to induce regenerative responses in the human body; namely, dentin bridges of improved quality and enhanced sealing of the pulp capped site. Depending on the purpose, CSMs products can be assigned into two clinically relevant groups: restorative cements used in VPT, i.e., ProRoot MTA, MTA Angelus, RetroMTA, Biodentine and TheraCal LC, and endodontic sealers, i.e., BioRoot RCS (Septodont, France). Differences between composition of each material are presented in Table 1.

### 3.1. Mineral Trioxide Aggregate

Mineral trioxide aggregate (MTA) is a bioactive cement pioneered by Torabinejad et al. in the early 1990s as an endodontic repair and root-end filling material [28] with favorable physical properties [29]. MTA has proven to induce mineralization beneath exposed pulp and have the potential of maintaining pulp vitality. Hence, the indications for the use of MTA have expanded considerably from its original use, and it has recently became a superior substitute for Ca(OH)_2_ in many other clinical applications, including direct [14,15,30,31] and indirect pulp capping, perforation repairs in roots or in furcations [32] and the apexification procedure [33,34,35]. The powder of MTA is a mixture of a purified Portland cement and bismuth oxide to provide radiopacity. The main constituent phases of cement are tricalcium and dicalcium silicate and tricalcium aluminate [36,37].

ProRoot MTA, having been available on the market for two decades, has been extensively studied and proven to be biocompatible. However, the long setting time and also high cost have urged the development of new types of MTA-based materials to overcome these drawbacks (Table 2). The alternative materials must meet the advantages of the ProRoot MTA, and also be more accessible, more cost effective and set in a shorter time. As an alternative, MTA Angelus was developed, offering the advantage of reduced final setting time—24–83 min [38,39], down from the original 228–261 min specific for ProRoot MTA [37,40]. Another of the recently introduced fast-setting calcium silicate cements is Retro MTA, with a final setting time of about 12 min [39]. Although a few studies evaluated and compared the biocompatibility of each of these products with that of Pro Root MTA, those finding are limited and not comprehensive. Characteristics of those materials were collected and presented in Table 1, Table 2 and Table 3.

The biocompatibility and sealing ability of MTA result from the dominant calcium ion released from the material which reacts with phosphates in tissue fluid, inducing hydroxyapatite formation (Table 3) [59]. Formation of this layer is a key characteristic responsible for the chemical seal between MTA and the dentinal walls, and cannot be referred to as a genuine bonding process [60].

The first formulation of MTA was grey, but due to the reported discoloration of teeth [61], an altered chemical composition was presented to the market (Table 1). The chemical component of iron is absent in white MTA, although discoloration is still observed and remains one of the major drawbacks of the material [62,63]. Tricalcium and dicalcium silicate react when mixed with water to produce calcium silicate hydrate and calcium hydroxide (Table 1). Despite the high clinical advantages of MTA cement, there were always some limitations which prevented clinicians from using it in on a daily basis. The major ones being a long setting time (up to 284 min) [44,64], handling difficulties, discoloration of the remaining tooth structure [65] and the presence of heavy metals in the powder [66]. The aforementioned properties of MTA are listed in Table 1 and Table 2.

When compared with Ca(OH)_2_ in a randomized clinical trial, confirmatory evidence emerged for the superior performance of MTA as a DPC agent when evaluated in a practice-based research network [31]. In this study, the probability of failure at 24 months was 31.5% for Ca(OH)_2_ and 19.7% for MTA. A review of the few clinical assessments in 9–10-year observation revealed 92.5–97.96% success for the teeth pulp-capped with MTA [57,58]. Clinical success rates for compared materials are presented in Table 2.

In addition, MTA is less toxic, easier to use in pulp capping procedures and causes less pulpal inflammation than Ca(OH)_2_ [67]. A histological study confirmed that the application of MTA has a direct effect upon regeneration potential of the dental pulp and is associated with increase in TGF-β1 secretion from pulp cells (Table 3) [68]. This factor directs the progenitor cells’ migration to the material-pulp interface and stimulates their differentiation to odontoblastic cells secreting reparative dentin; thus it affects the quality of the induced hard barrier. Further histological study confirmed that the formation of calcified hard tissue after pulp capping with fast-setting MTA (RetroMTA) is not particularly the product of genuine odontoblast differentiation and lacks characteristics of “regular dentine” [69]. These results suggest that the formation of calcified tissues may be more appropriately regarded as a reparative process more than a genuine regeneration response. Hence, it can be concluded that regular dentine was not regenerated, and because of the limited bioactive potential of pulp-capping material (RetroMTA), it cannot be used in regenerative dentistry. Moreover, in contrast to calcium hydroxide, mineral trioxide aggregate (WhiteProRoot®MTA, Dentsply Sirona, York, PA, USA) and Biodentine (BD) exhibit favorable metabolic activity and promote the same desired cellular response, resulting in higher clinical success rate and less tunnel defects [70]. In terms of dentin bridge formation, based on the micro-CT imaging technique, the MTA group shows more a regular, homogenous reparative dentin layer with uniform thickness, when compared with the Biodentine group. These data indicate that both MTA and BD induced hard tissue barriers and that MTA induced dentin with better characteristics (Table 3) [71]. Therefore, MTA is the material of choice in direct pulp capping [70,72]. 

Other key factors for successful pulp capping procedures are the ability of the bioactive material to seal to the tooth structure, the bond strength between the pulp capping material and restorative properties (Table 2). Recent studies suggest that placement of composite used with a two-step etch and rinse adhesive (E&R) over white MTA performed significantly better than an all-in-one system in terms of bond strength [76]. Other studies concluded that the highest bond strength was obtained when the E&R adhesive was used after 24 h resulting in shear bond strength (SBS) of 7.3 ± 1.49 MPa [77,78]. Supplementary surface treatment protocols were investigated to reliably asses SBS after final composite restoration (Table 3). Additional silanization (Silane, Ultradent, South Jordan, UT, USA) was recommended after treating the surface of ProRoot MTA with 9% hydrofluoric acid (HF) for 90 s before the application of dentin bonding agent (DBA) for higher bond strength [82]. Further research also suggested air abrasion of MTA Angelus surface after 72 h with 50 µm Al_2_O_3_ particles to achieve higher bond strength to the resin composite compared to specimens treated with 37% phosphoric acid (16.98 ± 4.24 MPa and 11.40 ± 3.19 MPa, respectively) [54]. Although the manufacturers of ProRoot, MTA Angelus and Retro MTA claimed short setting times (165 min, 15 min and 1.5 min, respectively), studies showed adequate setting only after at least 7 days to acquire proper surface properties (Table 2 and Table 3) [83,84]. Additionally, evidence exists that MTA continues to mature up to 1 year beyond the setting time with impacts on its mechanical integrity and hence SBS values [85]. This research also puts forward the importance of leaving MTA to mature before the application of the overlying restoration to prevent bacterial infection.

#### Novel Mineral Trioxide Aggregate Restorative Cements

Alternation in material characteristics has recently led to development of new generation MTA-based cements; namely, Neo MTA Plus (Avalon Biomed Inc., Houston, TX, USA) and the iRoot (Innovative BioCeramix Inc., Vancouver, BC, Canada) products family. Both materials share the same clinical applications in vital pulp treatment, but only a few studies are available to assess their use in clinical practice [89,90,91,92]. Neo MTA Plus was developed to be used in pulpotomies without the risk of discoloration due to elimination of bismuth oxide. The radiopacifying agent was replaced by tantalum oxide, which provides a radiopacity value of 3.76 ± 0.13 mm Al and does not exert any effect on hydration [89,92]. What is important is that the final setting time of Neo MTA Plus was proven to be prolonged up to 315 ± 5 min [89]. Moreover, compared to MTA Angelus, NeoMTA Plus showed better apatite formation, higher crystallinity and higher Ca/P, but a lower CO_3_/PO_4_ ratio, which might result in increased bioactivity [90]. Further in vivo and in vitro studies are required to substantiate such claims.

Addressing the difficult handling of MTA, the iRoot products are available in different consistencies, what could offer an advantage of choosing suitable one for each clinical application. Namely, iRoot BP (IBC, Burnaby, BC, USA) is deposited on preloaded syringes, while the iRoot BP Plus is available in jars and with a thicker consistency, and the iRoot FS was especially developed to set faster with final setting time of 57.0 ± 2.7 min [83]. A systematic review concluded that iRoot BP and iRoot BP Plus are biocompatible materials that enhance human dental pulp cells’ proliferation, migration, mineralization and dentinal bridge formation in pulp capping procedures [93]. These findings coincided with those obtained in another study, stating that iRoot BP Plus showed superiority over calcium hydroxide as a pulpotomy agent [94].

There are only a few studies on novel CSMs, as the scientific evidence is in general focused on materials that have been available for a longer time, such as ProRoot MTA and later Biodentine. To determine their clinical efficacy, further studies are needed.

### 3.2. Biodentine

As a response to the disadvantages of MTA, a new tricalcium silicate-based cement Biodentine (Septodont, France) was released in 2011. This comparatively new biomaterial is claimed to possess properties similar to MTA and is currently being explored for vital pulp therapy procedures. BD was designed as a permanent, biocompatible [95] dentin substitute that could be applied in one session for final composite restoration with the sandwich technique or in the whole volume of the cavity for an observation period before the final restoration. The manufacturer indicates the setting time to be between 9 and 12 min [96]; however, it was proven to set finally after 45 min (Table 2) [43].

BD is available in the form of a capsule containing powder composed of tricalcium silicate, dicalcium silicate, zirconium oxide, calcium carbonate, calcium oxide and iron oxide. A single capsule containing 0.7 g of powder is mixed for 30 s in a mixing device at a speed of 4000–4200 rpm with exactly five drops of liquid containing calcium chloride which act as an accelerator [97]; a hydrosoluble polymer functioning as a water reducing agent; and water (Table 1). The setting accelerator improves its handling properties and strength and mitigates the risk of partial material loss and alteration of the interface when compared to MTA [13,22,45]. Concerning drawbacks of the material, radiopacity is significantly lower than MTA Angelus (Table 2) despite the presence of zirconium oxide [98]. Radiopacity also gradually decreases with time, which causes difficulties in long-term radiographic observations.

The interactions of BD with hard and soft tissues in both the direct and indirect capping procedure lead to a marginal sealing and provide protection to the underlying pulp by inducing tertiary dentin synthesis and remineralization. Based on the calcium (Ca^2+^) and hydroxide (OH^−^) ion release from material, it may be concluded that tricalcium silicate materials such as BD may be preferable for IPC (Table 3) [99].

Marginal sealing is provided by micromechanical retention due to penetration of Biodentine into the dentin tubules forming tag-like structures, and represents similar bond strength to dentine compared toMTA (ProRoot MTA) [49,100]. However, those findings are inconsistent with results of other studies suggesting that Biodentine is superior to MTA in terms of sealing ability [101,102,103].

When compared to MTA, improvements in BD properties, such as setting time, mechanical qualities and initial cohesiveness, led to widened range of applications, including endodontic repair and vital pulp therapy. On account of its advantages, BD has recently become a preferable agent for both direct and indirect pulp capping procedures. Biodentine, compared with the previous golden standard Ca(OH)_2_, is mechanically stronger, less soluble and produces a tighter seal [100].

Similarly, another study was conducted to evaluate the clinical response of pulp-dentin complex after DPC with MTA and BD in carious teeth. Over a 6-month observation, MTA and BD showed success rates (subjective symptoms, pulp sensibility tests and radiographic appearance) of 91.7% and 83.3%, respectively [104]. Biodentine and MTA have comparable success rates when used as direct pulp capping or pulpotomy material in permanent mature teeth with carious exposure (Table 2). The success rate of the VPT decreased from overall 96% after 1-year follow-up to 93.8% at the 3-year follow-up (Biodentine—91.7% and MTA—96.0%) [105]. A randomized clinical trial was conducted to investigate the outcome of the DPC of permanent young teeth with Biodentine; it showed no failures after 12 months, while both calcium hydroxide and MTA had a 13.6% failure rate after the same time period [106]. Other studies also support claims of Biodentine’s and MTA’s superiority over calcium hydroxide in terms of success rate in pulp capping procedures [107,108]. Another study reported that the success rate of DPC with BD is 90.9% in patients younger than 40 and 73.8% in patients 40 or older [109]. It is worth emphasizing that patient’s age significantly influences the clinical outcome of this vital pulp therapy and needs to be taken into consideration when choosing successful treatment.

Based on a cell/tissue culture model, it can be concluded that materials used in pulp capping procedures directly affect the regeneration potential of the dental pulp by modulating the secretion of factors such as TGF-β1 [68,110]. Biodentine applied directly onto pulp, induces reparative dentine formation resulting in complete dentin bridge formation, absence of an inflammatory pulp response and layers of well-arranged odontoblasts and odontoblast-like cells observed after 6 weeks [108]. There is ample evidence for the positive effects of BD on vital pulp cells, for stimulating tertiary dentin formation and for early formation of reparative dentin [111]. Biodentine had a similar effect on dentin bridge formation to MTA (Table 3) [71,112,113]. In contrast to another study [46], a clinical trial in adults showed complete dentin bridge formation in 100% of Biodentine cases, compared to 11% and 56% in TheraCal® and ProRoot® MTA, respectively [114]. In terms of quality of induced dentin, MTA exceeded BD, although concerning completion of the dentin bridge, BD had better results. Therefore, those reports are inconclusive in choosing the most reliable pulp capping material. Nevertheless, in the later stages of differentiation and mineralization of dentin bridge, Biodentine had been observed to have a better performance. That is attributed to a notable increase in alkaline phosphatase expression and calcium nodule formation compared to the MTA characteristics [70]. The antibacterial properties of BD and MTA can refer to the high alkaline pH of these materials, which has an inhibitory effect on the growth of microorganism and causes disinfection of dentin (Table 3) [115].

Due to unsatisfactory aesthetics and physical properties of Biodentine, there is a need for an overlying material with final restoration, usually a composite. Yet, despite the possibility of immediate restoration placing after initial set, there is statistically significant increase in SBS when bonding with Biodentine after a 72-h maturation period [86]. Moreover, Biodentine presents a considerably lower SBS to composite than glassionomer cement (GI) even after a 2-week maturation period (Table 3).

Although Biodentine offers many advantages over MTA, it presented significantly lower SBS values than MTA to restorative materials, including composite (Filtek Z250, 3M, Saint Paul, MN, USA), compomer (Dyract XP®) and RMGI cement (Photac-Fil Quick Aplicap, 3M, Saint Paul, MN, USA) [116]. On the other hand, another study showed clinically acceptable scores and higher SBS of Biodentine compared to MTA when used with the methacrylate-based composite [53]. Inferior results obtained with Biodentine might be explained by the deficient intrinsic maturation, which can take over 2 weeks [87,88]. These results highlight the importance of leaving Biodentine and MTA to mature before the application of the overlying restoration for better clinical outcomes.

### 3.3. TheraCal LC

TheraCal LC (Bisco, Schaumburg, IL, USA) was introduced in 2011 to overcome poor bonding of CSMs to resins in final restorations. TheraCal LC is a light-cured calcium silicate-based material designed as both a direct and indirect pulp capping material that facilitates the immediate placement of final restoration.

A material’s ability to remineralize tooth’s structure is associated with the resin formula of TheraCal LC that possesses calcium and hydroxide ion release properties. The bioavailability of calcium ions released form TheraCal LC is proven to be in the concentration range for potential stimulatory activity for dental pulp and odontoblasts, although still significantly lower than in the case of Biodentine [117,118,119]. The hydration process of Theracal LC was found to be incomplete due to the limited moisture diffusion within the material [118]. Thus, no calcium hydroxide is produced, and less calcium ion leaching is recorded, resulting in an inferior remineralization potential compared to Biodentine. The absence of calcium hydroxide in set TheraCal LC suggests that calcium ions released from this material are not in the hydroxide form. Hence, it can be concluded that the presence of a resin matrix modifies the setting mechanism and calcium ion kinetics of TheraCal LC, resulting in lower calcium-releasing ability (Table 3) [120]. In vitro study reported that the CSMs (Biodentine, ProRoot MTA) induced remineralization of artificially demineralized dentine at a definitely higher speed and intensity than TheraCal LC [121].

The material seals the pulp capping site despite contact with dentinal or pulpal fluids, as its solubility is lower than ProRoot MTA, MTA Angelus and Biodentine (Table 2) [26]. Additionally, dentinal fluids play a crucial role in the release of calcium and hydroxide ions supporting the sealing capacity of the induced apatite. TheraCal LC exhibited superior sealing ability and comparable interfacial microleakage to MTA and Biodentine, showing better performance overall [122].

Lack of cytotoxicity and biocompatibility are the significant factors referring to pulp capping agents which directly affect the clinical outcome. Based on evaluation of pulpal responses in dog partial pulpotomy cases, complete dentin bridge was observed only in 33% of specimens [123]. It was found that TheraCal LC produced the least favorable pulpal responses compared to both ProRoot MTA and RetroMTA. Overall, research reported that TheraCal specimens had lower quality calcific barrier formation, extensive inflammation and less favorable odontoblastic layer formation (Table 3). Those findings were attributed to the presence of acrylic monomer Bis-GMA in the material. However, it should be noted that Bis-GMA was not detected, despite being listed in the safety data sheet provided by the supplier [124]. Presence of resin in the pulp capping agent which may remain unpolymerized is often associated with adverse pulpal reactions that lead to pulp toxicity and inflammation. The study designed to investigate the consequences of adding resins to tricalcium silicates by comparative analysis showed that TheraCal is toxic to pulp fibroblasts and has a higher inflammatory effect and a lower bioactive potential than Biodentine [125]. Those findings coincide with another study which reported that the reparative capacity of TheraCal LC is inferior to Biodentine [126]. TheraCal LC results showed dentin bridge formation with mild chronic inflammation, reduced dentin bridge thickness and a higher inflammatory score, which may be attributed to the hydration properties of TheraCal LC (Table 3). Moreover, despite that the photopolymerization of TheraCal LC is associated with low heat generation, it could still potentially induce adverse pulpal effects when used in pulp capping procedures [127].

TheraCal LC exhibits higher bond strength values than Biodentine when layered with either composite or glassionomer cement [128,129]. To enhance SBS, E&R adhesives are recommended when placing composite restoration on TheraCal LC (Table 2 and Table 3) [81].

Although sufficient bioactivity, superior handling properties and superior quality of bonding with the final overlaying restoration could justify the use of TheraCal LC as the IPC agent, further in vitro and in vivo studies are required. Furthermore, TheraCal cannot be recommended for DPC [125].

### 3.4. ACTIVA BioACTIVE

ACTIVA BioACTIVE-BASE/LINER (Pulpdent, USA) was launched in 2014 claiming the strength, aesthetics and physical properties of composites and increased release and recharge of calcium, phosphate and fluoride in comparison with glassionomer (GI). On the other hand, it has been proven that the fluoride ion release of ACTIVA is lower than that of the conventional GI (Keta Molar Quick Aplicap, 3M ESPE, Saint Paul, MN, USA) and also than that of RMGI cement (VitremerTM, 3M-ESPE, Saint Paul, MN, USA) [130,131]. The results indicate the ACTIVA BioACTIVE does uptake fluoride and re-release it, which could offer decreased incidence of secondary caries. At this time, the ion-releasing ability of these materials has not been proven to protect from caries at restoration margins.

The company markets this product as a “light-cured resin-modified calcium silicate” (RMCS) combining uncompromised attributes of both composite and GI [132]. Despite claimed bioactivity, the manufacturer recommends the use of ACTIVA BioACTIVE products in cases without pulpal involvement and ACTIVA BioACTIVE-BASE/LINER only in cases of IPC.

Comparing to both MTA and Biodentine, ACTIVA BioACTIVE represents a favorable setting time with no delay placing final restoration (Table 2). The material has three setting mechanisms; it cures with low intensity light for 20 s per layer and has both glass-ionomer (acid-base reaction) and composite self-cure setting reactions (Table 1). The anaerobic, self-cure (best under oxygen barrier; e.g., glycerine) setting-time is 3 min. It is also advisable to allow the material to self-cure for 15–20 s before light curing.

The bioactive properties of ACTIVA BioACTIVE products are based on a mechanism whereby the material responds to pH cycles and plays an active role in releasing and recharging of significant amounts of calcium, phosphate and fluoride [131]. Some authors have suggested that ability to release biologically active ions is more accurately termed “biointeractivity” and is a prerequisite for a material to be bioactive [117]. These mineral components are responsible for stimulating the formation of mineralized hard tissue. As calcium ions play a key role in the material-induced proliferation and differentiation of human dental pulp cells, they also stimulate the formation of a connective apatite layer and seal at the material-tooth interface. ACTIVA exhibited the potential to stimulate biomineralization at the same level as MTA, Biodentine and TheraCal LC on the basis of releasing the same amount of Ca and OH ions (ionic supplemented conditions) (data not shown in Table 3) [133].

Despite the fact that ACTIVA BioACTIVE products are RMGICs, the laboratory and clinical findings indicate that the self-adhesive ability of the material is not elucidated [52]. The aforementioned hypothesis was confirmed in a 1-year clinical follow-up of posterior restorations made with ACTIVA BioACTIVE Restorative, indicating a very high initial failure rate [134]. According to the manufacturer, no additional pretreatment of the capped site is required (Table 3). Studies have reported overall higher microleakage of cavities restored with ACTIVA BioACTIVE if no previous etching was performed nor an adhesive applied, compared to cavities restored with resin composite [135,136]. Similar observations were presented in the study where bond strength measurement of ACTIVA BioACTIVE Restorative after 28 days was not possible due to loss of restorations if no pretreatment was performed or if dentine was etched [52]. Under the conditions of this study, the conclusion can be drawn that self-adhesive property of ACTIVA BioACTIVE products is nonexistent; thus, it exhibits an insufficient protection of pulp in VPT (Table 3).

Considering the material’s characteristics, the application of ACTIVA BioACTIVE in direct and indirect pulp capping might be justified. Nevertheless, to this day there is insufficient evidence and there have been too few clinical studies to support its reliability in vital pulp therapies. Moreover, the resin in pulp capping materials such as ACTIVA BioACTIVE BASE/LINER would presumably shift the regeneration-inflammation balance towards the latter. As in the other light-cured tricalcium silicate, incomplete resin photopolymerization may lead to free monomers’ release, and consequently to pulpal toxicity [137]. However, to accurately assess ACTIVA BioACTIVE’s reparative potential or influence on the vital pulp in pulp capping procedures, further in vitro and in vivo studies are necessary. It can be stated that compared with calcium silicates, resin-containing materials are not fully consistent with the spirit of DPC. On the contrary, calcium silicate materials such as Biodentine and MTA shift the balance towards regeneration, resulting in successful clinical outcomes [138]. However, a recent in vivo study concluded that ACTIVA BioACTIVE BASE/LINER exhibited excellent biocompatibility and healing for rat subcutaneous tissues in comparison with CSMs (MTA-HP and iRoot BP Plus) [139]. Nevertheless, further evidence is needed to substantiate such claims.

## 4. Conclusions

Development of minimally invasive biologically based therapies aimed at preservation of the pulp vitality remains the key theme within contemporary clinical endodontics. The present findings confirm that both MTA and Biodentine are reliable materials in the matter of inducing dentin bridge formation while keeping a vital pulp in both direct and indirect pulp capping procedures [71,104,108,112,140]. This review also reports Biodentine’s superiority in relatively easier manipulation, lower cost and faster setting when compared to MTA with comparable or even outstanding clinical outcomes (Table 2). High biocompatibility and excellent bioactivity further go in favor of this dental replacement material, although more long-term clinical studies are needed for a definitive evaluation of Biodentine as a pulp capping agent.

Future in vitro and in vivo studies are necessary to validate the clinical importance of the new generation of light-cured resin-modified calcium silicates; namely, TheraCal LC and ACTIVA BioACTIVE BASE/LINER. In addition, more studies are needed to support use of these materials in VPT other than indirect pulp capping. RMGIC’s superior handling properties, quality of bonding with final overlaying restoration and the possibility of immediate restoration placement, might result in more predictable treatments from both a histological and a clinical perspective. Therefore, those materials should constitute the objects of future studies, especially in terms of cytotoxicity, quality of induced dentin bridge and protocols for higher bond strength to tooth structure and final restoration.

## Figures and Tables

**Table 1:** General information on pulp capping agents.

Property	Material					
	MTA			Biodentine	TheraCal LC	ACTIVA BioACTIVE BASE/LINER
	ProRoot MTA	MTA Angelus	RetroMTA			
Release Date	1999	2001	2014	2011	2011	2014
Composition	Powder: tricalcium silicate, icalcium silicate, tricalcium aluminate, bismuth oxide, gypsum Liquid: water	Powder: tricalcium silicate, dicalcium silicate, tricalcium aluminate, silicon oxide, potassium oxide, aluminum oxide, sodium oxide, iron oxide, calcium oxide, bismuth oxide, magnesium oxide, insoluble residues of crystalline silica Liquid: water	Powder: calcium carbonate, silicon dioxide, aluminium oxide, calcium zirconia complex Liquid: water	Powder: tricalcium silicate, dicalcium silicate, calcium oxide, calcium carbonate, zirconium oxide, iron oxide Liquid: calcium chloride, water-soluble polymer, water	Light-curing single paste: resin bis-phenyl glycidyl methacrylate (BisGMA) & polyethylene glycol dimethacrylate (PEGD) modified calcium silicate filled with CaO, calcium silicate particles (type III Portland cement), Sr glass, fumed silica, barium sulphate, barium zirconate	Diurethane dimethacrylate. bis (2-(methacryloyloxy) ethyl) phosphate, barium glass, ionomer glass, polyacrylic acid/maleic acid copolymer, dual-cure chemistry, sodium fluoride, colorants
Color	White	White	White	White	White	Tooth-shade
Mixing	0.5 g pouches of powder + pre-measured unit dose of water (mixed manually)	Powder + liquid (mixed manually)	0.3 g pouches of powder + 3 drops of water (mixed manually)	0.7 g capsule of powder + 5 drops of liquid (30 s; 4000–4200 rpm)	Dispensed directly from a flowable syringe (no mixing)	Two-paste system dispensed directly from an automix syringe
Setting reaction	Hydration reaction				Light-cure (20 s)	3 setting mechanisms: - self-cure - light-cure (20 s) - acid-base reaction
	MTA + water→calcium hydroxide + calcium silicate hydrate			Tricalcium silicate + water→hydrated calcium silicate gel + calcium hydroxide		

**Table 2:** Key properties of bio-inductive materials used in vital pulp treatment (VPT)—clinical manipulation and performance.

Property		Material					
		MTA			Biodentine	TheraCal LC	ACTIVA BioACTIVE BASE/LINER
		ProRoot MTA	MTA Angelus	RetroMTA			
Discoloration of tooth structure		+	+	+	-	-	-
Final setting time (min)		261 ± 21 [40] 228.33 ± 2.88 [37]	24.0 [38] 48.3 ± 4 [41,42] 83.66 ± 17.61 [39]	12.66 ± 3.05 [39]	45.0 [43] 85.66 ± 6.03 [37]	Immediate	2.5–3.0
Single visit treatment		-	-	-	+	+	+
Handling		+	+	+	++	+++	+++
Consistency		Granular, initial looseness			Uniform, putty-like	Flowable	Flowable
Cytotoxicity		NA	NA	NA	NA	Observed	Observed
Radiopacity (mm Al)		6.4–8.5 [44]	4.5–5.96 [44]	4.07 ± 0.20 [45] 3.01 ± 0.09 [46]	1.5–4.1 [43,44] 2.79 ± 0.22 [47]	2.17 ± 0.17 [47]	NA
Solubility at 24 h (%)		1.735 ± 0.328 [48] 10.89 ± 0.48 [26]	29.55 ± 2.35 [26]	1.447 ± 0.201 [48]	11.83 ± 0.52 [26]	2.75 ± 1.04 [26]	NA
Bond strength to dentine (MPa)	After 24 h	0 [49]	NA	1.15 ± 0.32 [50]	1.01 ± 0.13 [50]	0.44 ± 0.20 [50] 0.09 ± 0.20 [51]	NA
	After 7 days	0.85 ± 1.42 [49]	NA	NA	9.75 ± 2.19 [49]	NA	NA
	After 14 days	4.96 ± 4.54 [49]	NA	NA	9.34 ± 1.01 [49]	NA	23.7 ± 17.8 [52] after 28 days after DBA application
SBS to composite after 24 h (MPa)	Methacrylate-based composites	8.9 ± 5.7 [53]	11.40 ± 3.19 [54] SE DBA (7th generation)	4.71 ± 2.35 [55] SE DBA (7th generation)	17.7 ± 6.2 [53]	19.3 ± 8.4 [56]	NA
	Silorane-based composites	7.4 ± 3.3 [53]	NA	NA	8.0 ± 3.6 [53]	3.6 ± 2.5 [56]	
Clinical success rate in VPT (%)		80–97 [31,57,58] (up to 9–10 years)			73–96	NA	NA
Cost of single package		€45 for 0.5 g	€50 for 1 g	€14 for 0.3 g	€10 for a 0.7 g capsule	€20 for a 1 g syringe	€90 for a 7 mL syringe
Approximate cost per application (€)		22.5	12.5	14	10	5	3.5

NA—not available; SE—self-etch; DBA—dentine bonding agent.

**Table 3:** Interfacial properties of bio-active materials.

Property		Material					
		MTA			Biodentine	TheraCal LC	ACTIVA BioACTIVE BASE/LINER
		ProRoot MTA	MTA Angelus	RetroMTA			
Marginal seal to dentine		○ Chemical and/or micromechanical adhesion ○ Penetration in dentinal tubules				○ Low SBS due to polymerization shrinkage ○ Poor chemical or micromechanical adhesion	○ Poor chemical or micromechanical adhesion due to lack of self-adhesive properties ○ Good seal after DBA application
pH Initially/Endpoint		9.93/8.00 [45] 12.48/11.56 [73] 10.99/7.20 [26]	10.48–9.45 [74] 11.71–10.57 [73] 11.31–8.94 [26]	9.93/7.9 [45]	11.98/11.16 [75] 11.63/9.21 [26]	10.66/9.85 [73] 8.54/8.00 [26]	8.00
Calcium release (ppm)		15.7–27.4 [26]	11.7–55.1 [26]	NA	18.0–95.3 [26]	12.6–34.2 [26]	NA
Pulp/dentine treatment		Rinse with 2.6–5.0% NaOCl	-	-	Hemostasis	Hemostasis	Lightly dry, DBA for higher SBS [52] (E&R DBA not required acc. to manufacturer)
Response of the pulp		○ Non-inflammatory reaction, ○ Increase in TGF-β1, ○ Non-toxic to pulp cells, ○ Favourable odontoblastic layer formation			○ Non-inflammatory reaction, ○ Increase in TGF-β1, ○ Non-toxic to pulp cells, ○ Well-arranged odontoblasts	○ Mild chronic inflammation, ○ Toxic to pulp fibroblasts, ○ Less favourable odontoblastic layer formation	○ Biointeractive, ○ Toxic to pulp cells due to resin component?
Hard tissue barrier quality		○ Regular ○ Homogenous ○ Uniform thickness ○ Lacks characteristics of natural dentine			○ Complete dentin bridge formation ○ Regular ○ Uniform thickness	○ Low quality calcific barrier ○ Inferior dentin bridge formation ○ Reduced dentin bridge thickness	NA
Surface treatment before composite placement	Recommended by manufacturer	37% H_3_PO_4_ (15 s) DBA	-	-	DBA	DBA	ACTIVA BioACTIVE RESTORATIVE or DBA + composite [52]
	Recommended by research	E&R DBA [76,77,78]			2-SE DBA [79,80]	E&R DBA (higher SBS) [81]	NA
		9% HF (90 s), Silane [82]	(after 72 h) 50-µm Al_2_O_3_ (15 s, 7 mm distance) [54]				
Maturation period		≥7 days [83,84] 1 year [85]			72 h [86] >2 weeks [87,88]	-	NA

NA—not available; E&R—etch and rinse; 2-SE—two-step self-etch; DBA—dentine bonding agent; SBS—shear bond strength.
